# THE APPENDICITIS INFLAMMATORY RESPONSE SCORE FOR ACUTE APPENDICITIS: IS IT IMPORTANT FOR EARLY DIAGNOSIS?

**DOI:** 10.1590/0102-672020220002e1686

**Published:** 2022-09-16

**Authors:** Vitor Steil DEBONI, Matheus Ignácio ROSA, André Carminati LIMA, Agnaldo José GRACIANO, Christian Evangelista GARCIA

**Affiliations:** 1 São José Municipal Hospital, General Surgery Residency - Joinville (SC), Brazil;; 2 São José Municipal Hospital, Department of Surgery - Joinville (SC), Brazil.

**Keywords:** Appendicitis, Clinical Diagnosis, Tomography, Apendicite, Diagnóstico Clínico, Tomografia

## Abstract

The use of Appendicitis Inflammatory Response clinical score in patients with suspected acute appendicitis makes the diagnosis more objective and accurate.

**AIMS:**

The aim of this study was to prospectively compare two groups with suspected acute appendicitis, analyzing the number of imaging tests requested, waiting time in the emergency department, until definition of conduct, as well as the sensitivity and specificity of this diagnostic method.

**METHODS:**

This is a prospective randomized study comparing 55 patients submitted to clinical-radiological diagnosis according to the routine of the service (control group), with another 55 patients submitted to the Appendicitis Inflammatory Response score flowchart (intervention group).

**RESULTS:**

Waiting time for defining the intervention group’s conduct was 1.5 h shorter than the control group (p=0.02). Computed tomography was performed in 42 patients in the control group, compared with 25 in the intervention group (p=0.001). The impact of the flowchart based on the Appendicitis Inflammatory Response score of the cases compared to the control group was the reduction of appendectomies with a normal-appearing appendix from 5 to 1 and an increase in the exclusion of appendicitis diagnoses. The use of the Appendicitis Inflammatory Response score resulted in a diagnostic specificity of 92%, compared to 29% in the control group.

**CONCLUSIONS:**

The use of the Appendicitis Inflammatory Response score reduced the waiting time for the diagnosis of acute appendicitis, decreased the number of imaging tests, and increased diagnostic specificity of the disease.

## RESUMO - RACIONAL

A utilização do escore clínico Appendicitis Inflammatory Response em pacientes com suspeita de apendicite aguda torna o diagnóstico mais objetivo e preciso.

**OBJETIVOS:**

Comparar prospectivamente dois grupos com suspeita de apendicite aguda, analisando o número de exames de imagem solicitados, o tempo de espera no Pronto-Socorro, até a definição da conduta, bem como a sensibilidade e especificidade desse método diagnóstico.

**MÉTODOS:**

Estudo prospectivo randomizado comparando 55 pacientes submetidos ao diagnóstico clínico-radiológico de acordo com a rotina do Serviço (grupo controle), com outros 55 pacientes submetidos ao fluxograma do escore Appendicitis Inflammatory Response (grupo intervenção).

**RESULTADOS:**

O tempo de espera para definir a conduta do grupo intervenção foi 1,5 hora menor do que o grupo controle (p=0,02). A tomografia computadorizada foi realizada em 42 pacientes do grupo controle, em comparação com 25 do grupo intervenção (p=0,001). O impacto do fluxograma baseado no escore Appendicitis Inflammatory Response dos casos em relação ao grupo controle foi a redução de apendicectomias com apêndice de aparência normal de 5 para 1 e um aumento na exclusão de diagnósticos de apendicite. O uso do escore Appendicitis Inflammatory Response resultou em especificidade diagnóstica de 92%, comparado a 29% no grupo controle.

**CONCLUSÕES:**

A utilização do escore Appendicitis Inflammatory Response reduziu o tempo de espera para o diagnóstico de apendicite aguda, diminuiu o número de exames de imagem e aumentou a especificidade diagnóstica da doença.

## INTRODUCTION

Acute appendicitis (AA) is a common cause of abdominal pain at all ages, with a lifetime prevalence of one case in seven people^15^. In the initial phase, the symptoms can be vague and nonspecific, especially in women^15^. The final diagnosis is usually based on clinical history, physical examination, and related laboratory and imaging tests^6^ ^,^ ^17^ ^.^

Early diagnosis of AA is essential for reducing morbidity and mortality associated with advanced stages of the disease. Therefore, imaging tests such as ultrasound (US) and tomography are often used to clarify the diagnosis of AA^2^ ^,^ ^3^. However, performing routine imaging tests for patients with abdominal pain can mean an increase in hospital costs and the length of stay of patients in the emergency care units, until the definitive conduct^13^ ^,^ ^14^.

Consequently, several diagnostic scores have been developed to aid in the diagnosis of AA, derived from systematic clinical analyses^5^ ^,^ ^7^ ^,^ ^12^. These scores aim to reduce uncertainty by standardizing the collection and interpretation of clinical and laboratory data^8^. Risk stratification using clinical scores has the potential to improve the diagnosis of AA and the management of hospital resources^6^ ^,^ ^18^.

Among the clinical scores described, the Appendicitis Inflammatory Response (AIR) was superior to the Alvarado score, most used in clinical studies, with superior accuracy in the evaluation of patients with suspected AA, reducing the need for imaging tests and the number of hospital admissions for low-risk patients without compromising investigation safety^1^ ^,^ ^9^.

Some studies have evaluated the use of scores to aid decision-making in suspected cases of AA, providing agility in follow-up or surgical indication, in cases with low-risk or high-risk scores, respectively^4^ ^,^ ^10^. However, the use of the flowchart suggested by the consensus of AA is still not a reality among emergency physicians.

The objective of this study was to evaluate the effectiveness of the use of the AIR score against a suspicion of AA in a referral hospital for the care of these patients and to analyze the use of imaging tests and the length of stay of the patient in the emergency unit.

## METHODS

This is a prospective randomized study comparing patients admitted to the emergency department of São José Hospital Municipal from Joinville, with suspected AA. Patients were randomly divided into two groups: control or intervention, according to a computerized list generated by the Random Allocation Software program^11^.

The **control** group included patients managed according to the routines and procedures currently used in the emergency surgery service, for the investigation of suspected cases of AA.

**Figure 1 -:**
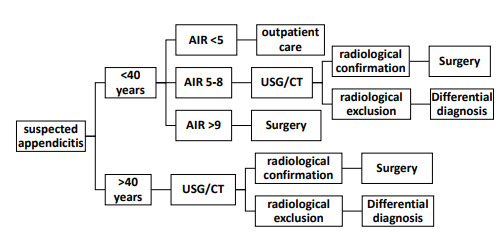
Flowchart used in the research.

AIR: Appendicitis Inflammatory Response; USG: Ultrasonography/CT: computed tomography.

The **intervention** group followed the flowchart (Figure 1) adapted from Saverio et al.^4^. Patients under 40 years of age, classified as low risk, were followed up and were instructed to return to the emergency department at any time or routinely for reassessment at the Hospital’s General Surgery Outpatient Clinic.

The AIR score on signs and symptoms found in patients with clinical suspicion of AA is described in Table 1.

**Table 1 -:** Score used in patients with clinical suspicion of acute appendicitis to determine the Appendicitis Inflammatory Response score.

Symptoms, signs, and laboratory tests		Score
Vomiting		1
Pain in the right iliac region		1
Tenderness and rigidity in the right iliac region	Light	1
	Moderate	2
	severe	3
Temperature >38.5°C		1
	Moderate-severe	4
Leukocytes (x10^9^)	≥10 and <15	1
	≥15	2
Neutrophils %	≥70 and <85	1
	≥85	2

The expected sample for the study was 100 patients, based on a retrospective analysis of appendectomies in the past 3 years of the Service. Data collection took place between April and September 2021, totaling 110 cases.

Inclusion criteria were patients, over 16 years old, seen at the hospital emergency department with suspicious clinical symptoms or a definite diagnosis of AA. Exclusion criteria were younger than 15 years.

Data collected include age, sex, comorbidities, information on imaging test results (computed tomography CT scans and ultrasonography - USG), length of stay of the patient in the emergency department (beginning of care until discharge or admission), and macroscopic and histopathological analysis of the appendix of the cases submitted to surgical treatment.

For statistics, analysis of variance or nonparametric Kruskal-Wallis test was used to compare quantitative variables, and the association between qualitative variables was evaluated using the chi-square test.

This research was approved by the Research Ethics Committee of São José Municipal Hospital, Joinville-SC, under number 5362, with informed consent.

## RESULTS

The flowchart used in this research is shown in Figure 1.

Most patients were young adults between 16 and 40 years (71%), with a mean age of 26.3 years. There was a slight predominance of males (51.8%), without statistical significance (p=0.7).

**Figure 2 -:**
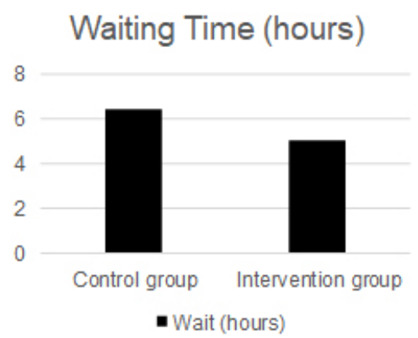
Waiting time in hours of the patient in the emergency department, in the control group and intervention group.

The patient’s waiting time in the emergency department, until being admitted for surgery or being discharged for outpatient return, was on average 6.4 h in the control group and 5 h in the intervention group with statistical significance (p=0.03) (Figure 2).

A greater number of imaging tests were performed in patients in the control group. US was performed in 23 patients in the control group compared to 22 indications in the intervention group, without statistical significance (p=0.2) (Table 2). CT was performed in 42 patients in the control group compared to 25 indications in the intervention group, with statistical significance (p=0.001) (Table 3).

**Table 2 -:** Abdominal ultrasound performed or not in patients in the control and intervention groups (p=0.2).

USG	CONTROL GROUP	INTERVENTION GROUP
Did not perform	32	33
Perform	23	22

USG: Ultrasonography.

**Table 3 -:** Abdominal computed tomography performed or not in patients in the control and intervention groups (p=0.001).

CT	CONTROL GROUP	INTERVENTION GROUP
Did not perform	13	30
Perform	42	25

CT: computed tomography.

Surgical treatment was not indicated in 20% of patients in the intervention group versus 3% in the control group. None of the patients in whom surgery was contraindicated were readmitted to the emergency department for investigation or treatment of AA.

The number of appendectomies without histopathological changes was higher in the control group compared to that in the intervention group (9 vs. 1.5%). The diagnostic sensitivity of both the groups was 100%, with specificity being 29% in the control group and 92% in the intervention group (Tables 4 and 5).

**Table 4 -:** Sensitivity and specificity of the clinical diagnosis of acute appendicitis in the control group (sensitivity 100%; specificity 29%).

CONTROL GROUP	APPENDICITIS	NORMAL APPENDIX
Positive clinical diagnosis	48	5
Negative clinical diagnosis	0	2

**Table 5 -:** Sensitivity and specificity of the Appendicitis Inflammatory Response score in the diagnosis of acute appendicitis in the intervention group (sensitivity 100%; specificity 92%).

INTERVENTION GROUP	APPENDICITIS	NORMAL APPENDIX
Positive AIR flowchart	42	1
Negative AIR flowchart	0	12

AIR: Appendicitis Inflammatory Response.

Patients who scored low on the AIR score or had differential diagnoses such as urolithiasis, pelvic inflammatory disease, and gastroenteritis were followed up on an outpatient basis with treatment aimed at these etiologies (Table 6).

**Table 6 -:** Differential diagnosis of three patients treated with suspected acute appendicitis.

Differential diagnosis	Number of cases
Urinary lithiasis	1
Acute cholecystitis	1
Right colon tumor	1

## DISCUSSION

CT was the most accurate examination, diagnosing AA in 77.6% of patients, compared to 51% of those who underwent US in both groups. CT was necessary to diagnose AA in 13 patients who underwent US with inconclusive results^16^.

All patients in the **intervention** group who received high risk according to the AIR underwent surgical treatment without performing additional tests, with a histopathological diagnosis of AA. In the **control** group, all patients at high risk according to the AIR underwent CT before appendectomy and no appendices without histopathological changes were observed in patients at high risk. Therefore, the AIR score flowchart proved to be safe in indicating surgical treatment without performing imaging tests for these patients.

A critical analysis regarding the AIR score, observed during data collection, was the subjectivity in the abdominal defense criterion. The original article describing AIR does not set out objective criteria for scoring. This item receives a score from 1 to 3, according to the examiner’s assessment. However, a definition in the final conduct was noticed by the weight of this score. A more objective criterion would facilitate the application of the score.

It was possible to determine a difference in the waiting time of patients in the emergency department until the definition of the AA diagnosis. Patients in the control group waited 1.4 h longer than patients in the intervention group. Therefore, the flowchart applied decreased by more than 1 h, until the final conduct, hospital discharge, or surgical treatment.

As for the final outcome of the cases, there were no false-negative results in the diagnosis of AA in the **control** group, as in the flowchart based on the AIR score of the **intervention** group, therefore giving a sensitivity of 100%. Regarding specificity, there was a difference between the group (29 control vs. 92% intervention), demonstrating the effectiveness of the flowchart based on the AIR score to safely rule out the diagnosis of appendicitis, reducing the frequency of appendectomies with the appendix showing a normal appearance.

## CONCLUSION

The flowchart used in this study proved to be effective in reducing the patient’s waiting time in the emergency department and also in reducing complementary examinations for the diagnosis of abdominal pain suspected of AA. The reduction in the number of requested imaging tests observed in the intervention group implies lower hospital costs.
